# Development of High-Performance Mullite Steel-Pouring Brick

**DOI:** 10.3390/ma19091819

**Published:** 2026-04-29

**Authors:** Mingyang Huang, Yuefeng Qi, Xiaocheng Liang, Guoqi Liu, Benjun Cheng

**Affiliations:** 1 School of Energy Science and Engineering, Central South University, Changsha 410083, China; 243912066@csu.edu.cn; 2 Henan Zhongyuan Special Steel Equipment Manufacturing Co., Ltd., Jiyuan 454640, China; 15639136210@163.com; 3 State Key Laboratory of Advanced Refractories, Shanghai University, Shanghai 200444, China; lxc2025@shu.edu.cn (X.L.); liuguoqi@shu.edu.cn (G.L.); 4 School of Materials Science and Engineering, Shanghai University, Shanghai 200444, China

**Keywords:** clay, zircon powder contents, steel-pouring brick

## Abstract

The effects of different clay addition amounts and zircon powder contents on the properties of steel-pouring brick were studied using bauxite particles, sintered mullite powder, and other materials as the main raw materials. The results showed that with the decrease of clay content, the bulk density of the samples changed slightly, but the cold modulus of rupture, compressive strength, hot modulus of rupture, and high-temperature volume stability were improved; the addition of zircon powder did not significantly change the basic properties, such as the bulk density of the brick, but significantly increased the high-temperature flexural strength. Therefore, under the addition of 5 wt.% clay and 1.5 wt.% zircon powder were used for the application. The results show that samples with this formula have good performance, and the surface of the cast steel is smooth. Therefore, the optimization of the mullite steel-pouring brick formula can adopt the strategy of the synergy effect of clay and zircon to improve its comprehensive performance.

## 1. Introduction

Steel-pouring brick is a key refractory component used to guide and distribute the flow of high-temperature molten steel in the iron and steel industry. Its main function is to construct a stable channel during molten steel pouring (especially the continuous casting process) or transfer to ensure that the molten steel flows according to the preset path [1], while protecting the equipment body from direct scouring and erosion by high-temperature molten steel. The working condition of steel-pouring brick is direct contact with molten steel at 1500–1700 °C, experiencing severe temperature fluctuations (close to room temperature before molten iron flows, and high temperature during scouring). The core requirement is to withstand the extreme environment in steel casting, which has strict requirements on its material and performance. Therefore, it should have the following properties: 1. to avoid cracking, it needs excellent thermal shock resistance [2,3]; 2. good high-temperature resistance; 3. to resist the mechanical wear of high-speed flowing molten steel and the chemical corrosion of refractory materials by slag (such as CaO, SiO_2_) in molten steel, it needs excellent slag corrosion resistance [4,5,6]; and 4. to prevent molten steel or slag from penetrating into the brick body, leading to structural spalling or molten steel contamination, it should have excellent impermeability and low porosity [7,8].

At present, common steel-pouring bricks are mainly composed of alumina (Al_2_O_3_) and SiO_2_. Alumina materials have a high melting point and can withstand the high-temperature effect of molten steel [9]. However, a small amount of impurities, such as Fe_2_O_3_ and CaO, in steel-pouring bricks will reduce the melting point of Al_2_O_3_, leading to liquid phase sintering at high temperatures and reducing its high-temperature resistance. In terms of thermal shock resistance, alumina is a typical brittle material with poor toughness at both room temperature and high temperature, lacking the “plastic deformation” ability to relieve thermal stress [10,11]. Therefore, with the increasing development requirements of the steel casting industry, higher requirements have been put forward for the performance and material of steel-pouring bricks. At present, the shortcomings of alumina-based steel-pouring bricks, such as insufficient toughness, easy permeability, and insufficient corrosion resistance, have gradually emerged in use, and more advanced schemes are urgently needed to improve the performance of steel-pouring bricks [12,13].

Compared with traditional alumina steel-pouring bricks (Al_2_O_3_), mullite steel-pouring bricks (3Al_2_O_3_·2SiO_2_) show multiple performance advantages in the complex service environment of the iron and steel industry. On the one hand, the formation of mullite in the matrix of steel-pouring bricks can increase the expansibility of the material, which can partially offset the volume shrinkage caused by the formation of the glass phase by impurities, thereby improving the refractoriness under load. On the other hand, mullite has low expansion and excellent chemical stability, and its thermal conductivity is between that of high-alumina bricks and lightweight refractory materials, which can slow down the temperature gradient inside the brick body, avoid thermal shock fracture caused by excessive local temperature difference of the material, and, thus, improve the thermal shock stability of the material and accurately match the core performance requirements of steel-pouring bricks under steelmaking conditions [14,15].

Wahsh, M.M.S. et al. prepared alumina–mullite–zirconia composites using a reaction bonding method, using alumina and zircon sand as raw materials and sintering at 1300 °C, 1400 °C, and 1500 °C. The results showed that the specimens sintered at 1500 °C for 2 h exhibited better densification, mechanical properties, and thermal shock resistance than those sintered at lower temperatures. This improvement was mainly attributed to the fact that high temperature promotes a sufficient reaction of raw materials, accelerates liquid phase formation and particle sintering, effectively reduces internal pores, and enhances structural integrity [16]. The study by De Oliveira et al. further validated the advantages of high-temperature sintering. They sintered high-alumina refractory materials containing a zircon aggregate at 1600 °C for 5 h and found that the fracture energy of the specimens was significantly higher than that of the reference specimens sintered at conventional temperatures. Moreover, the coarse aggregate particles exhibited excellent thermal shock resistance, which was closely related to the full development of the mullite phase at high temperature and the good bonding between the zircon aggregate and the matrix [17]. In addition, the sintering temperature directly affects the phase evolution of the material. Liu, Z.Y. et al. found that when the sintering temperature increased from 1450 °C to 1560 °C, the main phases of the composite ceramics remained mullite and corundum, accompanied by a small amount of zirconia phase, and the bulk density continuously increased with rising temperatures, indicating that higher temperatures promote phase stabilization and densification [18]. Mecif, A. et al. pointed out that the formation reaction of zircon (ZrSiO_4_) begins at 1150 °C, and a high-temperature environment accelerates this reaction while affecting the final phase composition and pore structure [19]. Therefore, the effect of the zircon addition on material properties exhibits a double-edged sword characteristic: an appropriate amount can significantly enhance the comprehensive properties, while excessive amounts lead to property degradation. In terms of positive regulation, the transformation toughening effect of zirconia is particularly prominent. Yildiz, B.K. et al. studied the effect of different volume fractions (0, 0.5, 1, 3, 5, 10, and 20) of zirconia on the flexural strength of alumina–zirconia composites. They found that when the zirconia volume fraction was 20%, the flexural strength of the composite reached a peak value of 435 ± 78 MPa, which is about 24% higher than that of pure alumina. This result confirms that an appropriate amount of zirconia can improve the mechanical properties of the material through the transformation toughening mechanism [20]. Meanwhile, zircon (zirconia) can also enhance the corrosion resistance and thermal shock resistance of the material. Aksel, C. et al. used a static crucible method and found that with increasing zirconia content, the wettability of the glass melt on the refractory material decreased. The undissolved zirconia particles formed physical and energy barriers, effectively preventing the penetration of molten glass into the material pores, thereby reducing the degree of corrosion [21]. De Oliveira et al. also confirmed that the formulation containing fine mullite and agglomerated zircon aggregate exhibited significantly better thermal shock resistance than the reference refractory material. Moreover, the lateral pores observed in the aggregate could act as buffers against thermal expansion, further improving thermal shock stability. On the negative side, excessive amounts of zircon (zirconia) lead to increased porosity and decreased high-temperature stability. Nasrollahnezhad, Rana et al. [22] conducted corrosion tests in a molten steel environment at 1480 °C, and found that pure zirconia specimens without additives began to decompose at 1650 °C, whereas the addition of 15 wt.% alumina accelerated the decomposition process, indicating that an excessively high zirconia content reduces the high-temperature stability of the material. The study by Mecif, A. et al. showed that when the zirconia addition exceeded 20 wt.%, the porosity of the final product increased significantly. This is mainly because the increase in zirconia content leads to a relative decrease in clay content, and the total amount of fluxing impurities (such as K, Fe, Ca, and Mn) in the clay decreases, affecting the sintering densification process and, thus, degrading the material properties. In addition, excessive zircon (zirconia) particles tend to agglomerate, leading to loose interparticle bonding and the formation of more pore defects, which become channels for corrosive media penetration, further reducing the corrosion resistance and mechanical properties of the material.

Based on the above review, most studies have focused on the relationship between alumina content and zirconia content in alumina–zirconia refractories, mainly investigating the effects of changes in the content of a single component on the microstructure and mechanical properties of the materials. Their research results show that the mechanical properties of the materials improve with the increase in zirconia content. However, an excessively high zirconia content and an excessive reduction in clay content will affect the sintering properties of the samples, such as an increase in porosity. Moreover, an excessively high zirconia content leads to relatively high costs in industrial production. In-depth research is still lacking in the following aspects: the effects of changes in the content of mullite powder, zircon powder, and clay on the properties of steel-pouring bricks; research on the high-temperature flexural resistance of the materials; and the effect of steel-pouring bricks in actual production. On the premise of ensuring the properties of steel-pouring bricks, it is necessary to explore the appropriate ratio among mullite powder, zircon powder, and clay. Therefore, this paper explores the influence of zirconia with a content of 0–3% on mullite steel-pouring bricks through component adjustment, and analyzes the steel-pouring bricks combined with microstructure and industrial verification. Therefore, by lowering the sintering temperature and using more common industrial raw materials, this paper aims to develop a low-cost, high-performance steel pouring brick to meet the needs of actual production.

## 2. Materials and Methods

### 2.1. Materials

This experiment was adjusted based on the actual formula of Zhenming Hi-Tech Refractory Co., Ltd. in Changxing City, Zhejiang Province, China. The main raw materials used were 68 bauxite particles (1–3 mm), sintered mullite powder (0–0.5 mm), 75 high-alumina powder (0–0.5 mm), and 70 high-alumina powder (0–0.5 mm).

The chemical composition analysis of 68 bauxite particles is as follows (68.5 wt.%Al_2_O_3_, 26.6 wt.%SiO_2_, 1.71 wt.%Fe_2_O_3_, 0.15 wt.%MgO, 2.62 wt.%TiO_2_, 0.15 wt.%K_2_O, and 0.1 wt.%Na_2_O). The chemical analysis of Guangxi white clay is as follows (32.06 wt.%Al_2_O_3_, 48.27 wt.%SiO_2_, 1.56 wt.%Fe_2_O_3_, 0.13 wt.%CaO, 0.21 wt.%MgO, 0.23 wt.%K_2_O, 0.1 wt.%Na_2_O, and 1.48 wt.%TiO_2_). Bonder is sulfite pulping waste liquor.

First, the effect of reducing the clay content on the material properties was studied, and the specific formula is shown in Table 1. Then, the effect of zircon powder on the material properties was studied, and the specific formula is shown in Table 2. All raw materials were purchased from Zhenming Hi-Tech Refractory Co., Ltd. in Changxing City, Zhejiang Province, China.

### 2.2. Experimental Procedures

First, the raw materials were accurately weighed according to the formula, uniformly mixed in a mixer-roller, and extruded into shape in a mold. The sintering temperature of the samples was 1420 °C. Specimens were prepared in accordance with the Chinese national standards GB/T 3001-2017 [23] and GB/T 5072-2023 [24]: (25 mm × 25 mm × 140 mm) for flexural (or compressive) strength tests. Specimens were prepared in accordance with the Chinese national standard GB/T 2997-2015 [25] for bulk density and porosity tests. Specimens were prepared in accordance with the Chinese national standard GB/T 3002-2017 [26]: (25 mm × 25 mm × 140 mm) for high-temperature flexural strength tests. Six samples are prepared for each formula and tested, and the average value of the six test results is taken.

Phase analysis was performed using an X-Ray Diffractometer (XRD, MiniFlex600, Rigaku, Kyoto, Japan). XRD patterns were acquired at ambient temperature using Cu-Kα radiation, with scanning conducted over a 2θ range of 5° to 90° at a rate of 10°/min. The microstructures of heat-treated samples (polished) were examined using Field Emission Scanning Electron Microscopes (FE-SEM, GeminiSEM 300, Zeiss, Oberkochen, Germany), both equipped with Energy Dispersive Spectroscopy (EDS) systems. All polished samples were sputter-coated with gold prior to FE-SEM observation.

## 3. Results

### 3.1. Phase Analysis

**Figure 1:**
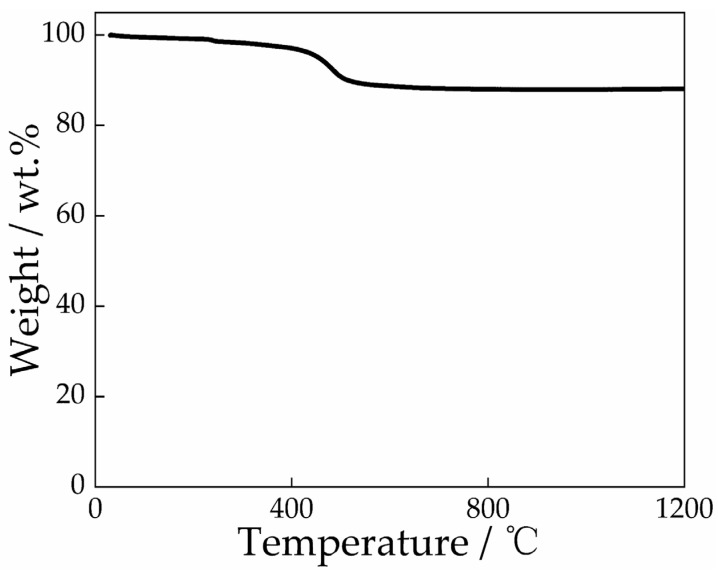
Thermo gravimetric analysis of Guangxi White Clay.

It can be seen from Figure 1 that, from room temperature to 400 °C, there is almost no obvious weight loss, indicating that the adsorbed water content in the sample is very low, with only a trace amount of surface water evaporating slowly and being barely detectable. From 400 °C to 500 °C, the sample undergoes a distinct, rapid weight loss stage, demonstrating that the dehydroxylation reaction of the sample occurs. From 500 °C to 1200 °C, the curve is completely stable with no additional weight loss, signifying that the structural water of the sample has been completely removed, and no further decomposition or weight loss reactions take place during the subsequent temperature rise.

**Figure 2:**
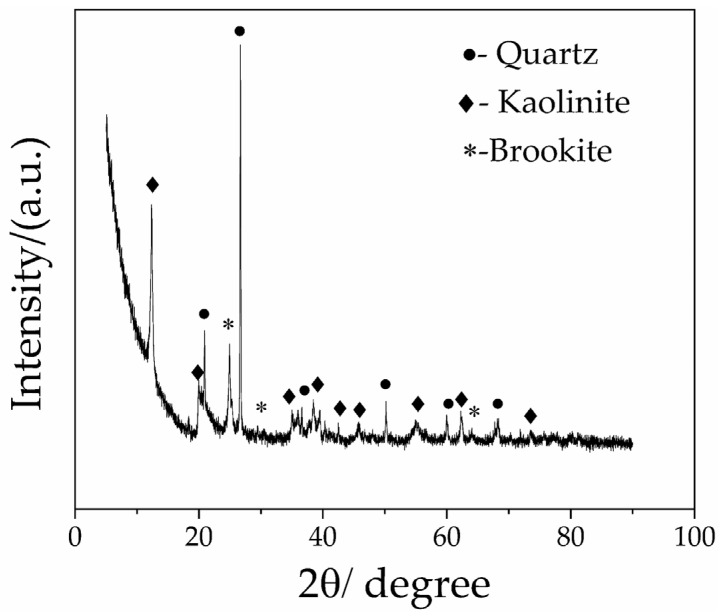
Phase analysis of Guangxi white clay.

X-ray diffraction (XRD) technology was employed to conduct a qualitative phase analysis on the Guangxi samples. The results show that the main crystalline phase in the samples is quartz, while the secondary crystalline phases are kaolinite and brookite, exhibiting the typical characteristic of coexistence of clay and siliceous in Figure 2. Among them, quartz exhibits the highest intensity of characteristic diffraction peaks, with sharp peak shapes and a narrow full width at half maximum, indicating that quartz is dominant in the sample, with a high crystallinity and intact crystal structure. As a typical clay, kaolinite shows clearly identifiable characteristic diffraction peaks with relatively gentle peak shapes, consistent with the general diffraction characteristics of clay minerals of slightly lower crystallinity and finer particle size, serving as the main clay phase of the white clay. The weak diffraction signal of brookite suggests that it exists as a trace-associated oxide phase in the sample.

**Figure 3:**
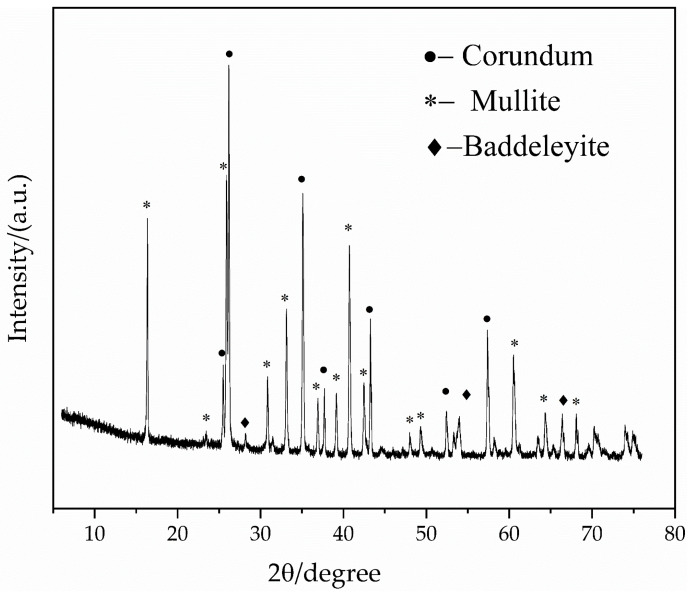
Phase analysis of sample (5 wt.% clay, 1.5 wt.% zircon powder, 48 wt.% 68 bauxite particles, 6 wt.% sintered mullite powder, 27 wt.% 75 high-alumina powder, 7 wt.% 70 high-alumina powder, and 0.5 wt.% bonder).

An X-ray diffraction (XRD) phase characterization was performed on mullite refractory brick samples with 1.5% zircon added. The results show that the main crystalline phase of the sample is corundum, the secondary crystalline phases are mullite and baddeleyite, and the phase composition is clear in Figure 3. Among them, the characteristic diffraction peaks of the corundum phase exhibited the highest intensity, sharp shape, and narrow full width at half maximum, indicating that it dominated the sample content with high crystallinity and well-developed crystals. Mullite served as the matrix mineral phase of the sample, with distinct characteristic diffraction peaks and regular peak shapes, demonstrating sufficient formation and stable structure of the mullite phase during the firing process. The diffraction peak intensity of the baddeleyite phase was relatively weak, clearly detectable, but relatively gentle, corresponding to the ZrO_2_ crystalline phase formed by the high-temperature decomposition and transformation of the added zircon, which was present in low content and dispersed distribution. The overall phase structure was dominated by the highly crystalline corundum, with mullite as the continuous matrix phase and baddeleyite uniformly existing as a trace modified phase. The three phases synergistically constituted the main crystalline phase framework of the refractory material, indicating complete phase transformation of the formula under the firing system and stable existence of zirconium components in the form of baddeleyite, which was conducive to improving the high-temperature performance and erosion resistance of the material.

### 3.2. Effect of Guangxi White Clay Content on Material Properties

**Figure 4:**
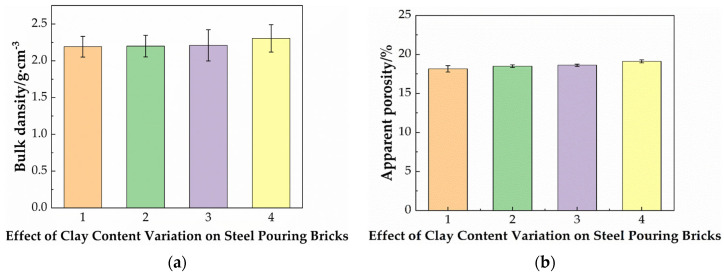
(**a**) The effect of the clay content on the bulk density; (**b**) apparent porosity of steel-pouring bricks.

Figure 4 shows the effect of the clay content on the bulk density and apparent porosity of steel-pouring bricks. It can be seen from the figure that with the decrease of the Guangxi white clay addition, the bulk density increases slightly, and the apparent porosity decreases moderately. This indicates that the Guangxi white clay has little effect on compactness.

**Figure 5:**
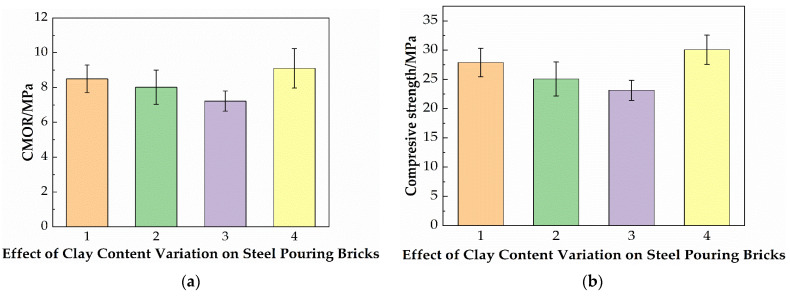
(**a**) The effect of the clay content on the cold modulus of rupture; (**b**) room temperature compressive strength of steel-pouring bricks.

Figure 5 shows the effect of the clay content on the cold modulus of rupture and room temperature compressive strength of the samples. It can be seen from the figure that with the decrease in clay content, the cold modulus of rupture and room temperature compressive strength decrease first and then increase. This is estimated to be related to the increase in the sintering temperature from 1390 °C to 1420 °C. Due to the increase in sintering temperature, the high clay content forms a more glassy phase during sintering, thus improving the room temperature strength of the material. After the clay content decreases, the amount of glass phase generated tends to be reasonable, which can ensure the bonding between hard particles at room temperature. The increase in the strength of sample No. 4 is due to the formation of a larger amount of the high-temperature mullite phase at a high firing temperature, which is beneficial to the high-temperature performance of the material.

Figure 5 shows the effect of the clay content on the high-temperature flexural strength and the linear change rates of samples. With the decrease of the Guangxi white clay addition, the high-temperature flexural strength of the samples gradually increases. The primary reason for the decrease of the glass phase and the bonding between particles no longer depends on a large amount of the softened glass phase, but mainly on intergranular sintering bonding or a small amount of the high-viscosity glass phase, which avoids the premature failure of grain boundaries and can effectively transmit bending stress, thereby improving the high-temperature flexural strength. After the reasonable reduction of the clay content, the sintering environment is more conducive to the precipitation and growth of mullite crystals, which can play a toughening effect. At high temperatures, these crystals can hinder crack propagation and further improve the high-temperature flexural strength of the brick [27].

**Figure 6:**
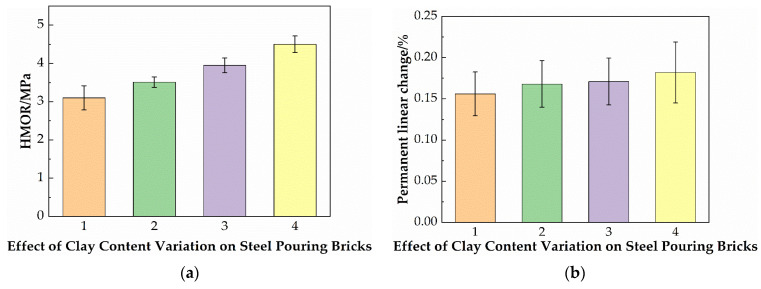
(**a**) The effect of the clay content on the high-temperature flexural strength; (**b**)the linear change rate after firing of steel-pouring bricks.

The test results of the linear change rate after firing are shown in Figure 6. It can be seen that the linear change rate gradually increases, but the change is not significant. This is because when the clay content is sufficient, the aggregate is wrapped by the clay liquid phase, and the particle rearrangement and densification are dominated by the liquid phase, which can promote the linear shrinkage rate of the material, thereby offsetting the linear expansion caused by the generation of mullite. With the clay content decreasing, the skeleton support effect of the aggregate becomes the dominant factor for volume stability, the volume proportion of the aggregate in the brick relatively increases, forming a more continuous and stable skeleton, and the load can be directly transmitted through the hard phase particles, resulting in a larger linear expansion due to the mullite generated.

### 3.3. Effect of Zircon Powder Content on Material Properties

**Figure 7:**
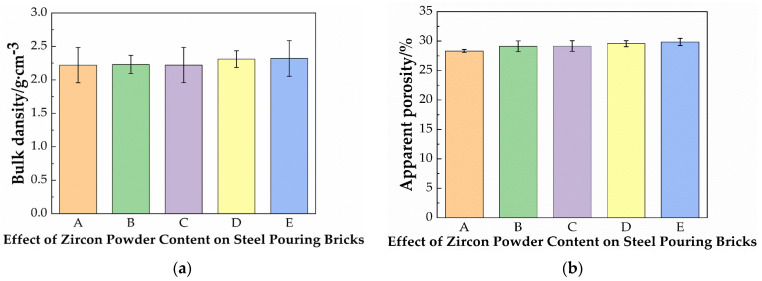
(**a**) Effect of the zircon content on the bulk density; (**b**) apparent porosity of steel-pouring bricks.

Figure 7 shows the effect of the zircon content on the bulk density and apparent porosity of specimens. It can be seen from Figure 7 that with the increase of the zircon powder content, the bulk density of the samples changes slightly. This is mainly because the specific gravity of the zircon raw materials is large, and the bulk density of the material tends to increase. On the other hand, the addition of zircon powder leads to a small amount of clay being added: zircon is an inert material, and the reduction in clay leads to a decrease in the glass phase. The apparent porosity of the material after sintering is large. The effects of the two mutually offset, resulting in little change in the bulk density after firing.

The apparent porosity of the samples is shown in Figure 7. With the addition of zircon powder, the apparent porosity gradually increases. This is because zircon powder hardly undergoes chemical reactions at the sintering temperature, and within a reasonable addition range, it can replace a portion of the clay materials, filling in the original pores. Furthermore, the decrease of clay powder will lead to the increase of mullite content and the decrease of glass phase, thus leading to the increase of apparent porosity.

**Figure 8:**
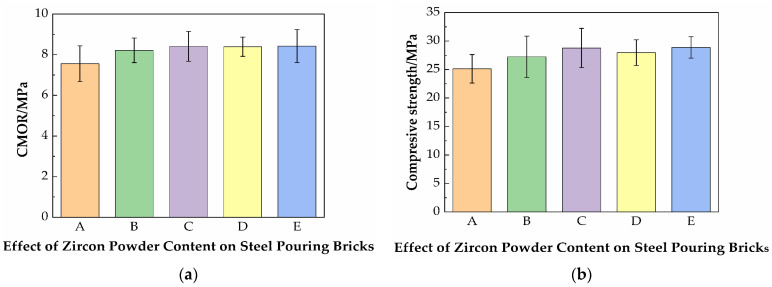
(**a**) Effect of the zircon content on the cold modulus of rupture; (**b**) room temperature compressive strength of steel-pouring bricks.

Figure 8 shows the effect of the zircon content on the cold modulus of rupture and room temperature compressive strength of specimens. With the addition of zircon powder, the cold modulus of rupture and room temperature compressive strength are gradually improved. This is mainly because the zircon powder is uniformly dispersed in the glass phase matrix formed by the high-temperature sintering of clay. A previous chemical analysis of the clay revealed the presence of calcium ions and other particles, which can promote the decomposition of zircon at low temperatures [28]. Furthermore, Al_2_O_3_ directly reacts with SiO_2_ in ZrSiO_4_ to form mullite and ZrO_2_, a process that lowers the decomposition temperature of ZrSiO_4_ and accelerates its decomposition [29]. This part was verified by the XRD results of the subsequent samples. These rigid fine particles bear part of the load, alleviate stress concentration in the matrix, and retard the initiation and propagation of cracks. In addition, more in situ mullite is revealed at the sintering temperature, thereby improving the flexural strength.

**Figure 9:**
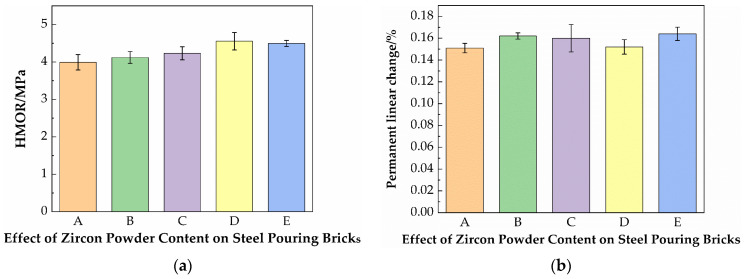
(**a**) Effect of the zircon content on the high-temperature flexural strength; (**b**) the linear change rate after firing of steel-pouring bricks.

Figure 9 shows the effect of the zircon content on the high-temperature flexural strength and linear change rate after firing of specimens. The test results of the high-temperature flexural strength of the samples are shown in Figure 9. With the addition of zircon powder, the high-temperature flexural strength first increases and then decreases, reaching the highest when the zircon addition amount is 1.5 wt.%. It is because the addition of zircon reduces the relative amount of clay added, and more mullite is generated at high temperatures. Thus, the high-temperature flexural strength and compressive strength are improved. When the zircon addition amount is 2%, the decrease of high-temperature flexural strength is estimated to be caused by large porosity or poor sintering at high temperatures, and the material undergoes insufficient sintering and exhibits high porosity. The underlying cause may be insufficient atomic diffusion, coupled with the synergistic effects of process factors including temperature, raw materials, liquid phase, and atmosphere, which results in impeded densification and the presence of residual porosity. With the addition of zircon powder, the linear change rate has fluctuated. This is because, in addition to the effect of clay variation on the formation of glass phase and mullite phase during sintering, the bonding or filling mode of zircon with the matrix phase also exerts a certain influence. The specific and detailed mechanism remains to be further investigated in subsequent research.

### 3.4. Microstructure Analysis

In actual use, the scouring and erosion of steel-pouring bricks by molten steel are the main causes of material damage. The improvement of high-temperature strength of the samples is the key factor to ensure the service condition of steel-pouring bricks and the casting quality of steel billets. Based on the research on the changes of clay and zircon contents, we believe that the effect of preparing steel-pouring bricks with the formula of 5 wt.% clay, 1.5 wt.% zircon powder, 48 wt.% 68 bauxite particles, 6 wt.% sintered mullite powder, 27 wt.% 75 high-alumina powder, 7 wt.% 70 high-alumina powder, and 0.5 wt.% bonder will be relatively ideal. The performance indicators of the prepared steel-pouring brick after testing are as follows: bulk density of 2.35g·cm^−3^, porosity of 17.8%, cold modulus of rupture of 8.9MPa, room-temperature compressive strength of 29 MPa, high-temperature flexural strength of 4.9MPa, and linear change rate after sintering of 0.17%. Therefore, a microstructural analysis is performed on sample D. The optimization of the sample’s microstructure analysis is as follows.

**Figure 10:**
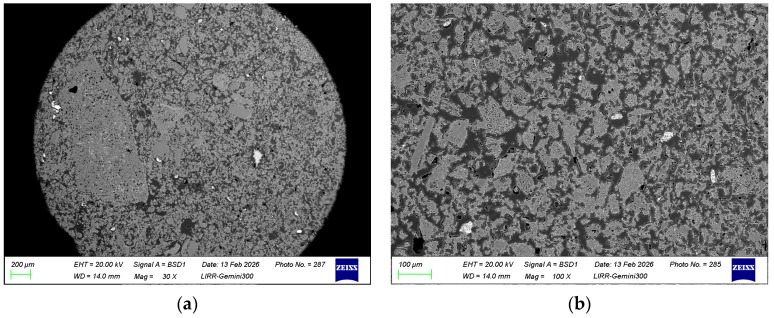
(**a**) The microstructure of the optimized sample; (**b**) the microstructure of the matrix of the optimized sample.

It can be seen from Figure 10 that the structure of the material is relatively dense, the fine powder in the matrix is distributed relatively uniformly, and the added zircon has decomposed.

**Figure 11:**
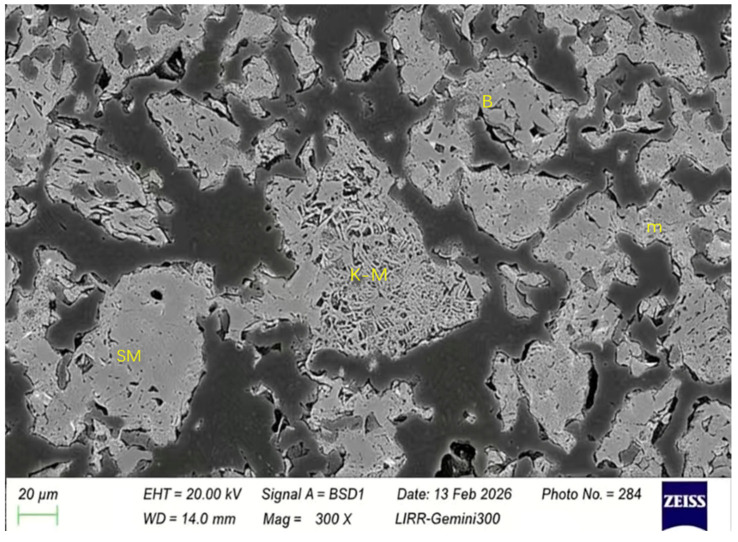
The microstructure of the mullite mineral phase in the matrix of the optimized sample.

**Figure 12:**
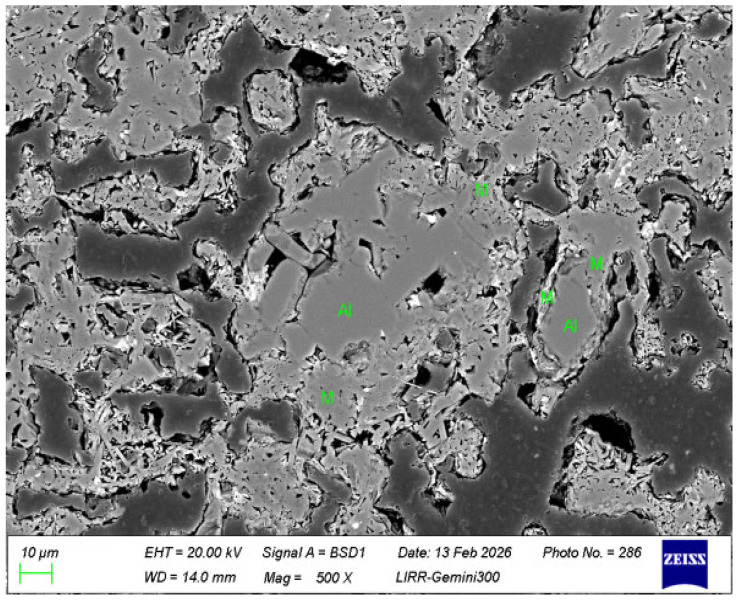
The microstructure of high-alumina powder in the optimized sample matrix.

It can be seen from Figure 11 that almost all the matrix of the material has turned into a mullite mineral phase or a mullite bonding structure. Among them, SM is the directly added mullite fine powder, K-M is the mullite fine powder formed from clay, and m is the mullite formed by the in situ reaction between silica in the clay or zircon and the aluminum oxide in the high-alumina material. B is a high-alumina powder, and the specific structure is shown in Figure 12.

It can be seen from Figure 12 that the center of the high-alumina powder remains a granular corundum crystal phase (Al), while the edges or surroundings have transformed into a columnar or acicular mullite mineral phase (M).

**Figure 13:**
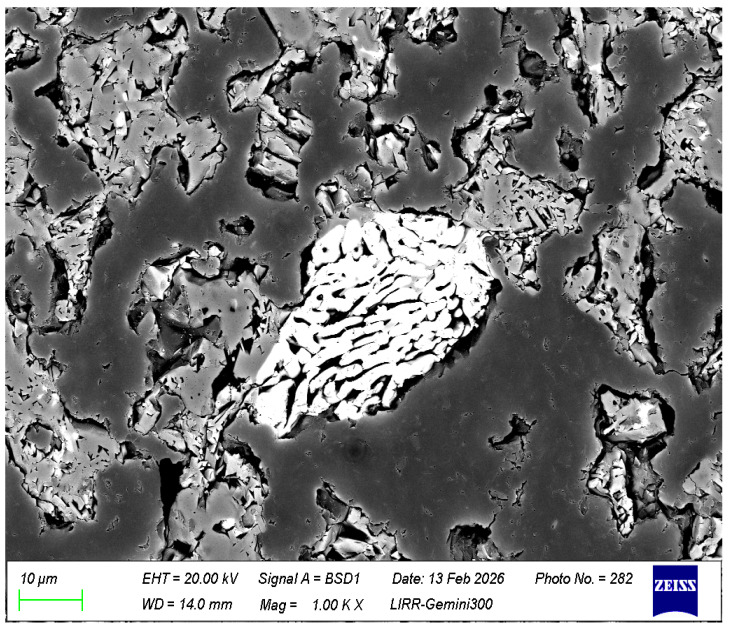
The microstructure diagram of zircon decomposition in the optimized sample matrix.

It can be seen from Figure 13 that zircon has been completely decomposed, forming fibrous or columnar monoclinic zirconia. Monoclinic zirconia has excellent high-temperature performance and dual toughening functions of microcracks and phase transformation, which greatly promote the improvement of the sample’s erosion resistance and thermal shock stability.

### 3.5. Industrial Experiment

This section was practically applied in a factory located in Changxing, Zhejiang Province. In this experiment, the tundish was lined with octagonal bricks and equally divided into two groups. Both groups were poured with the same batch of molten steel, with a total pouring amount of 12 tons and a pouring time of 20 min. A comparative test was conducted on the industrial site between product sample D, which was added with 1.5 wt.% zircon powder and 8.5 wt.% Guangxi white clay, and sample 3 without zircon powder but with 8.5 wt.% Guangxi white clay. It was found that the steel cast using the 1.5 wt.% zircon-added steel-pouring bricks featured a smooth surface and minimal residual steel slag.

**Figure 14:**
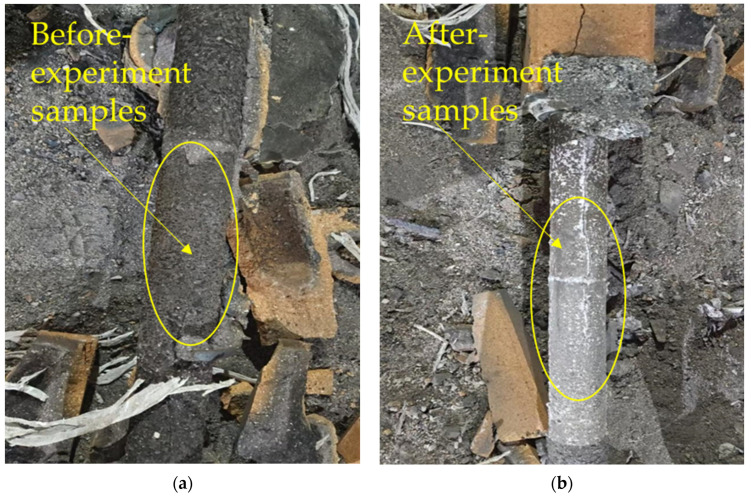
(**a**) Comparison between the sample without zircon powder added; (**b**) the sample with 1.5 wt.% zircon powder.

It can be seen from Figure 14 that the surface of the residual ingot before the test is rough, and the surface of the casting is black, indicating that the erosion of the steel-pouring brick using molten steel is serious, and the slag adhesion phenomenon is obvious. After the test, the surface of the ingot is smooth, and the casting quality is excellent.

## 4. Conclusions

This study systematically investigated the effects of the addition amount of Guangxi white clay and the content of zircon powder on the key properties of mullite steel-pouring bricks, providing data support for optimizing the formula of steel-pouring bricks and adapting to the harsh working conditions of steelmaking. The experimental results and discussions are as follows:

1. With the slight decrease in clay content, the cold modulus of rupture, room-temperature compressive strength, high-temperature flexural strength, and linear change rate of the material gradually increase, while the bulk density changes slightly. This is mainly due to the glass phase and mullite phase formed during sintering.

2. With the change of the zircon content, the cold modulus of rupture, room-temperature compressive strength, and high-temperature flexural strength of the material gradually increase, while the bulk density and linear change rate change slightly. This is mainly because the zircon powder is uniformly dispersed in the matrix of the glass phase formed by the clay during high-temperature sintering. These rigid fine powders bear part of the load, reduce the stress concentration of the matrix, and delay the initiation and propagation of cracks, thereby improving the mechanical properties of the material. On the other hand, more high-temperature mullite phases are generated at the sintering temperature, thereby improving the overall mechanical properties of the material.

3. A comparison was conducted between product sample D, which was added with 1.5 wt.% zircon powder and 8.5 wt.% Guangxi white clay, and Sample 3 without zircon powder but with 8.5 wt.% Guangxi white clay. The addition of zircon powder resulted in a slight increase in the apparent porosity and bulk density of sample D. However, its cold compressive strength increased by 20.9%, cold flexural strength by 16.2%, and hot flexural strength by 15.3% compared to the previous sample. The steel-pouring brick was produced based on the formula of 5 wt.% clay and 1.5 wt.% zircon powder has an excellent effect on the industrial experiment, and the casting quality is significantly improved.

In summary, the optimization of the mullite steel-pouring brick formula can adopt the idea of reducing clay and adding zircon, specifically improves the high-temperature strength and corrosion resistance potential, and ultimately achieves the precise matching between the comprehensive performance of the brick and the casting working conditions.

## Figures and Tables

**Table 1:** Formulations for the effect of clay content on material properties.

Raw Materials	1	2	3	4
68 bauxite particles, %	48	48	48	48
Sintered mullite powder, %	6.0	7.5	9.0	12.5
75 high-alumina powder, %	27	27	27	27
70 high-alumina powder, %	7.0	7.0	7.0	7.0
Guangxi white clay, %	11.5	10	8.5	5
Bonder, %	0.5	0.5	0.5	0.5

**Table 2:** Formulations for the effect of zircon content on material properties.

Raw Materials	A	B	C	D	E
68 bauxite particles, %	48	48	48	48	48
Sintered mullite powder, %	6.0	6.0	6.0	6.0	6.0
75 high-alumina powder, %	27	27	27	27	27
70 high-alumina powder, %	7.0	7.0	7.0	7.0	7.0
Guangxi white clay, %	11.5	11	10.5	10	8.5
Zircon powder, %	0	0.5	1	1.5	3
Bonder, %	0.5	0.5	0.5	0.5	0.5

## Data Availability

The original contributions presented in the study are included in the article. Further inquiries can be directed to the corresponding author.
